# Overexpression of DCLK1-AL Increases Tumor Cell Invasion, Drug Resistance, and KRAS Activation and Can Be Targeted to Inhibit Tumorigenesis in Pancreatic Cancer

**DOI:** 10.1155/2019/6402925

**Published:** 2019-08-05

**Authors:** Dongfeng Qu, Nathaniel Weygant, Jiannan Yao, Parthasarathy Chandrakesan, William L. Berry, Randal May, Kamille Pitts, Sanam Husain, Stan Lightfoot, Min Li, Timothy C. Wang, Guangyu An, Cynthia Clendenin, Ben Z. Stanger, Courtney W. Houchen

**Affiliations:** ^1^Department of Medicine, University of Oklahoma Health Sciences Center, Oklahoma City, OK, USA; ^2^Department of Veterans Affairs Medical Center, Oklahoma City, OK, USA; ^3^Peggy and Charles Stephenson Cancer Center, Oklahoma City, OK, USA; ^4^Department of Oncology, Beijing Chaoyang Hospital, Capital Medical University, Beijing, China; ^5^Department of Cell Biology, University of Oklahoma Health Sciences Center, Oklahoma City, OK, USA; ^6^Department of Pathology, University of Oklahoma Health Sciences Center, Oklahoma City, OK, USA; ^7^Department of Digestive and Liver Diseases, Columbia University Medical Center, New York, NY, USA; ^8^Department of Medicine, University of Pennsylvania Perelman School of Medicine, Philadelphia, PA, USA

## Abstract

Oncogenic KRAS mutation plays a key role in pancreatic ductal adenocarcinoma (PDAC) tumorigenesis with nearly 95% of PDAC harboring mutation-activated KRAS, which has been considered an undruggable target. Doublecortin-like kinase 1 (DCLK1) is often overexpressed in pancreatic cancer, and recent studies indicate that DCLK1+ PDAC cells can initiate pancreatic tumorigenesis. In this study, we investigate whether overexpressing DCLK1 activates RAS and promotes tumorigenesis, metastasis, and drug resistance. Human pancreatic cancer cells (AsPC-1 and MiaPaCa-2) were infected with lentivirus and selected to create stable DCLK1 isoform 2 (alpha-long, AL) overexpressing lines. The invasive potential of these cells relative to vector control was compared using Matrigel coated transwell assay. KRAS activation and interaction were determined by a pull-down assay and coimmunoprecipitation. Gemcitabine, mTOR (Everolimus), PI3K (LY-294002), and BCL-2 (ABT-199) inhibitors were used to evaluate drug resistance downstream of KRAS activation. Immunostaining of a PDAC tissue microarray was performed to detect DCLK1 alpha- and beta-long expression. Analysis of gene expression in human PDAC was performed using the TCGA PAAD dataset. The effects of targeting DCLK1 were studied using xenograft and Pdx1^Cre^Kras^G12D^Trp53^R172H/+^ (KPC) mouse models. Overexpression of DCLK1-AL drives a more than 2-fold increase in invasion and drug resistance and increased the activation of KRAS. Evidence from TCGA PAAD demonstrated that human PDACs expressing high levels of DCLK1 correlate with activated PI3K/AKT/MTOR-pathway signaling suggesting greater KRAS activity. High DCLK1 expression in normal adjacent tissue of PDAC correlated with poor survival and anti-DCLK1 mAb inhibited pancreatic tumor growth* in vivo *in mouse models.

## 1. Introduction

Pancreatic ductal adenocarcinoma (PDAC) has the worst prognosis of any major malignancy with less than an 8% 5-year survival rate and is the third leading cause of cancer-related deaths in the United States [1]. There are four major driver genes for pancreatic cancer: KRAS, CDKN2A, TP53, and SMAD4 [2–4]. KRAS mutations are harbored by 95% of PDACs and play a key role in PDAC tumorigenesis [5–7]. Active KRAS directs several downstream signaling pathways that play pivotal roles in proliferation, migration, invasion, and survival, which are the most important cellular mechanisms regulating PDAC tumorigenesis and metastasis.

Cells with cancer stem cell-like (CSC) properties have been identified in PDAC. These cells are often resistant to conventional chemotherapy and radiation therapy and as such may explain why current treatments do not cure PDAC or prevent recurrences. Doublecortin-like kinase 1 (DCLK1) is often overexpressed in pancreatic cancer and is coexpressed with other PDAC CSC markers, and recent studies indicate that DCLK1+ PDAC cells can initiate pancreatic tumorigenesis in the presence of mutation and inflammation [8, 9]. Functionally, we have also demonstrated that DCLK1 regulates key oncogenes, pluripotency factors, angiogenic factors, epithelial mesenchymal transition (EMT) related transcription factors, and pancreatic cancer xenograft growth which can be reversed by downregulating DCLK1 or inhibiting its kinase activity [10–13].

Many studies have reported targeting KRAS for PDAC treatment but it remains an undruggable target [4]. DCLK1 is strongly linked to KRAS-mutant cancer, as evidenced by its expression in tumor stem-like cells in multiple KRAS-mutant pancreatic cancer mouse models [14]. Moreover, Westphalen et al. demonstrated that Kras-mutant DCLK1+ tuft cells initiate cancer in the presence of inflammation in support of a CSC role in PDAC and also that DCLK1 forms a complex with KRAS [9]. On the molecular level, recent work using KRAS wild-type colorectal cancer cell line SW48 demonstrated that DCLK1 is transcriptionally induced by knock-in of KRAS G12D, G12V, or G13D, resulting in massive upregulation [15]. When KRAS is targeted with shRNA in these mutant SW48 cells, DCLK1 expression decreases in a dose-dependent fashion [15]. Similarly, when DCLK1 is targeted with siRNA, the expression of KRAS is decreased in a dose-dependent fashion [10, 11, 13]. Here, we report the role of DCLK1 in KRAS-PI3K-MTOR signaling pathway and its implications for chemoresistance and tumor growth. Importantly, our findings demonstrate for the first time that DCLK1 directly activates RAS and that DCLK1-targeted monoclonal antibodies can be used to inhibit PDAC tumor growth in xenografts and the KPC mouse model.

## 2. Materials and Methods

### 2.1. Ethics Statement

All animal experiments were performed with the approval and authorization from the Institutional Review Board and the Institutional Animal Care and Use Committee, University of Oklahoma Health Sciences Center and University of Pennsylvania Perelman School of Medicine. Mice were housed under controlled conditions, including a 12-h light-dark cycle, with ad libitum access to food and water.

### 2.2. Experimental Animals

Athymic nude mice were purchased from The Jackson Laboratory (Bar Harbor, Maine). Kras^LSL-G12D/+^;Trp53^LSL-R172H/+^;Pdx1-Cre (KPC) mice have been previously described [16] and were bred and maintained under two pathogen-free facilities at the University of Pennsylvania.

### 2.3. Analysis of DCLK1 Expression in Various Cancers

For DCLK1 mRNA expression levels in various cancer types, the Cancer Genome Atlas (TCGA) esophageal (ESCA), stomach (STAD), liver (LIHC), pancreas (PAAD), and colorectal (COADREAD) datasets were used. For DCLK1 protein expression levels in various cancer types and in normal tissues, the Human Protein Atlas (THPA) datasets were used [17–19].

### 2.4. Analysis of TCGA PAAD Data

The standard data run of The Cancer Genome Atlas PAAD dataset was downloaded and sorted for DCLK1 expression. Mann-Whitney U test was used for analysis and comparison of other gene expressions between these two groups (n=45 for each group).

### 2.5. Clinical Patient Characteristics

Only publicly available, deidentified data were accessed from TCGA for the analysis reported here. Basic characteristics of the PDAC patients used in the survival analysis are provided in Supplementary Tables S1 and S2.

### 2.6. Immunohistochemical Study of PDAC Tumor Tissue and Normal Adjacent Tissue

A pancreatic adenocarcinoma tissue microarray (US Biomax, HPan-Ade 180 Sur-02) containing 180 microsections including 60 paired tumor and normal adjacent tissues was immunostained with anti-DCLK1 antibody (Abcam, ab31704) following our previously described protocol [20]. Each stained tissue microsection was scored independently by two pathologists and based on percent of tissue demonstrating staining (1 for <10%-4 for > 60%) and staining intensity (1 for lowest intensity, 4 for highest intensity). The resulting scores were multiplied by each other to obtain a composite score.

### 2.7. Cell Culture and Establishing Stable Cell Lines

Human pancreatic cancer cell lines, AsPC-1 and MIA PaCa-2 (MP2), were obtained from ATCC and grown in Dulbecco's Modified Eagle's Medium with 4.5 g/L glucose and L-glutamine, without sodium pyruvate (Cellgro) supplemented with 10% fetal bovine serum (Sigma) at 37°C and 5% CO_2_. Lentivirus containing human DCLK1-AL cDNA sequence was constructed as described previously [21]. AsPC-1 and MP2 cells were infected with lentivirus to overexpress DCLK1AL-RFP fusion protein (AsPC-DCLK1 and MP2-DCLK1) or red fluorescent protein (AsPC-RFP and MP2-RFP) as control, and selected with puromycin to establish stable cell lines.

### 2.8. Drug Resistance Assays

Cells (5000 cells per well) were seeded into a 96-well tissue culture plate in triplicate. The cells were cultured in the presence of Gemcitabine (0, 12.5, 25, 50, 100, and 200 nM), everolimus (37.5 *μ*M), ABT-199, or LY-294002 (each at 0, 0.2, 0.4, 0.8, 1.6, 3.125, 6.25, 12.5, 25, 50, and 100 *μ*M) with DMSO as a vehicle control. 48 h after treatment, 10 *μ*l of TACS MTT Reagent (RND Systems) was added to each well and cells were incubated at 37°C for 4 h. Once dark crystalline precipitate became visible, 50 *μ*l of 266 mM NH_4_OH in DMSO [22] was added to the wells and placed on a plate shaker at low speed for 1 minute. The plate was measured at OD_550_ using a microplate reader. The OD value of each triplicate was averaged and the results were calculated as a percentage of the DMSO (vehicle) control +/- the standard error of the mean.

### 2.9. Matrigel Transwell Invasion Assay

Matrigel coated transwell assays (BD Biosciences) were prepared by soaking in serum-free media for 2 h at 37°C in a 24-well plate. MP2-RFP and MP2-DCLK1 cells (5000 cells/well) were seeded into each transwell in serum-free media in triplicate. Cell culture medium containing 10% FBS was added to the bottom of each well as chemoattractant and the cells were incubated for 22 h at 37°C. A cotton swab was used to scrape noninvasive/migratory cells off the top of transwell assays and the remaining cells were fixed with 100% methanol, stained with 0.1% crystal violet, and allowed to dry. After drying all invading cells were counted from each transwell at 4x magnificence.

### 2.10. In Vitro Spheroid Assay

MP2-RFP and MP2-DCLK1 cells (250 cells/well, n=6 per group) were seeded into an ultra-low attachment 96-well plate in RPMI containing 0.5% FBS and incubated at 37°C under 5% CO_2_ for 5 days. Medium without FBS was added on day 3 to prevent evaporation. On day 5, spheroids were manually counted under a light microscope at 10x magnification, and representative images were taken. Spheroids were defined as having at least 10 cells. Efficiency of spheroid formation was calculated by dividing the number of spheroids formed by the number of cells seeded.

### 2.11. Active RAS Pull-Down Assay

Both AsPC-RFP and AsPC-DCLK1 cells were cultured in serum-free medium overnight, followed by full growth medium (10% FBS) for 15 min in the presence of either DMSO or XMD8-92 (15 *μ*M). Cells were lysed and active RAS were analyzed using the Active Ras Pull-Down and Detection Kit (Thermo Scientific) based on the instruction.

### 2.12. Coimmunoprecipitation Assay

Both AsPC-RFP and AsPC-DCLK1 cells were lysed with Pierce IP Lysis Buffer (Thermo Fisher Scientific). The cell lysates were used for immunoprecipitation by incubating with anti-RAS antibody for 2h at room temperature, spinning down the precipitates with Protein A conjugated anti-mouse secondary antibody, washing 3 times with Pierce IP washing buffer, and eluting with gel loading buffer. The eluates were separated on a SDS-PAGE and subjected to western blot analysis with anti-DCLK1 antibody (Abcam, ab31704).

### 2.13. Western Blot Analysis

Total proteins of cell lysates were subjected to Western Blot analysis. The concentration of total proteins was determined by BCA protein assay. Equivalent amounts of total proteins were separated on a SDS-PAGE and transferred onto a nitrocellulose membrane. The membrane was blocked with 5% nonfat dry milk and probed with the primary antibody. The membrane was then incubated with IRDye 800CW-conjugated secondary antibody. The proteins were detected using Li-Cor Odyssey system.

### 2.14. Generation of a Human/Mouse Chimeric Antibody

DCLK1-targeted therapeutic monoclonal antibody (CBT-15mAb) and isotype control mAb were supplied in PBS (COARE Biotechnology). In addition, total RNA was isolated from monoclonal hybridoma cells secreting DCLK1 antibody (CBT-15); cDNA was synthesized using a primer downstream of the last variable region for heavy chain (HC) constant and light chain (LC) kappa constant. Each RT-reaction was subject to PCR using degenerate primer sets (USBIO, 11904-10A) to amplify all likely rearrangements. To create the human/mouse IgG chimeric antibody, PCR fragments from the above reaction were inserted into pFUSEss-CHIg-hG1 to express heavy chain and pFUSEss-CLIg-hK to express light chain kappa. Heavy chain was further cloned into pLenti CMV PURO DEST and light chain kappa was further cloned into pLenti CMV BLAST DEST. The expression plasmids constructed above were cotransfected along with packaging plasmids pMD2.G (Addgene), pMDL/RRE g/p (Addgene), and pRSV-Rev (Addgene) into 293T cells. Generation of the concentrated lentivirus was done as described previously [13]. Human 293T cells were infected with both concentrated viruses containing heavy chain and light chain and selected with puromycin and blasticidin (Sigma-Aldrich) to establish stable cell lines. The established cell lines expressing both heavy chain and light chain were expanded into a Bioreactor for production. The conditioned media were collected and purified using a Nab Protein L Spin column (Thermo Fisher Scientific) to produce CBT-15X mAB.

### 2.15. Xenograft Tumor Study

SW1990 or AsPC-1 pancreatic cancer cells (0.5x10^6^) in Matrigel were injected into the flanks of 8-week old athymic nude mice (n=6 for CBT-15 vs. isotype control groups and n=7 for CBT-15X vs. isotype control groups for both SW1990 and AsPC-1 cells) and allowed to grow to an average tumor volume of 100 mm^3^. Mice with xenografted tumors were injected intraperitoneally (*i.p*.) with CBT-15 mAb, CBT-15X mAb, or isotype control at 25 mg/kg twice per week. Tumor volume measurements were taken every other day using calipers. 30 days from the start of injections mice were killed and tumors excised, measured, and weighed.

### 2.16. KPC Mice Tumor Study

KPC mice with tumors measuring 50-100 mm^3^ were identified using ultrasonography. These mice were injected* i.p.* with CBT-15 mAb or IgG2a isotype control (n=4 for each group) at 25 mg/kg twice per week for four weeks. Tumors were measured by ultrasonography at baseline and once a week after intervention. Mice were killed after four-week treatment.

### 2.17. Statistical Analysis

All statistical analyses and figures were prepared using R v3.2, GraphPad Prism 6.0, SPSS Statistics 22, and Microsoft Excel. For nonparametric data the Mann-Whitney U test was used, and for parametric data Student's t-Test was used. Kaplan-Meier survival analyses were performed in GraphPad Prism 6.0. Cox regression analyses were performed using IBM SPSS Statistics 22. Heatmaps were generated using Genesis. A p-value of less than 0.05 was considered statistically significant for all analyses.

## 3. Results

### 3.1. DCLK1 Is Upregulated in Pancreatic and Other Cancer Types

**Figure 1:**
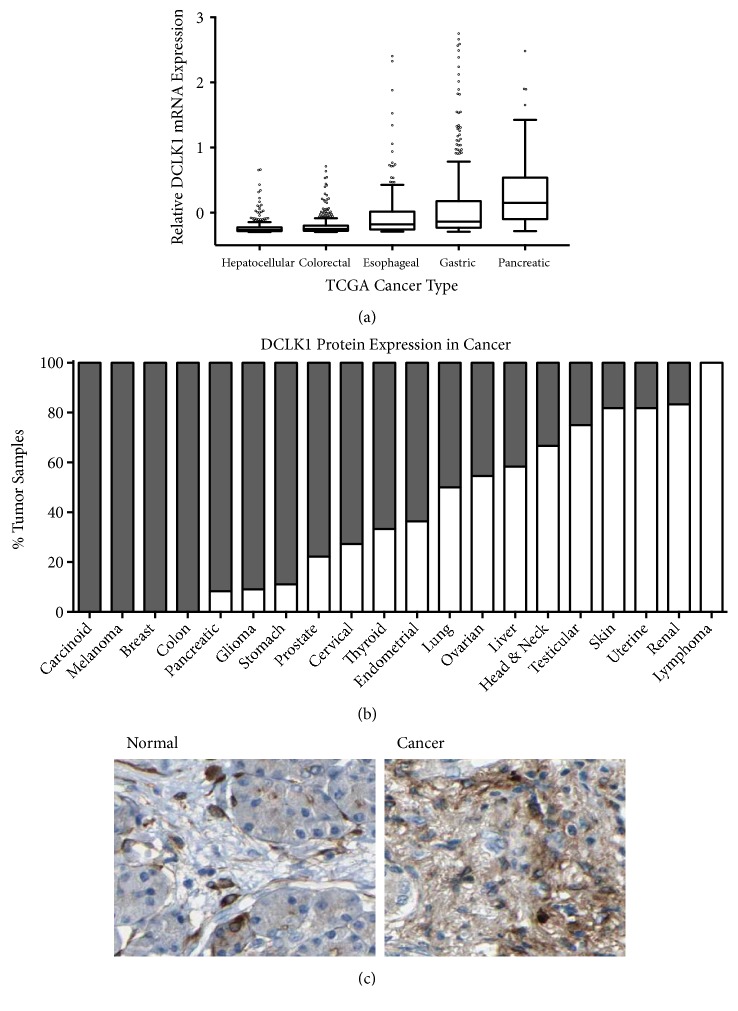
*DCLK1 expression is upregulated in pancreatic cancer and other cancer types*. (a) Relative DCLK1 mRNA expression levels were analyzed using the TCGA esophageal (ESCA), stomach (STAD), liver (LIHC), pancreas (PAAD), and colorectal (COADREAD) datasets. (b) Percentage of DCLK1 protein expression in various tumor tissues was analyzed using the Human Protein Atlas. (c) DCLK1 expression in the normal pancreas and cancer tissues was detected using anti-DCLK1 Ab immunostaining.

In order to assess DCLK1's gene expression pattern across gastrointestinal cancer types, we analyzed the TCGA esophageal (ESCA), stomach (STAD), liver (LIHC), pancreas (PAAD), and colorectal (COADREAD) datasets and found that pancreatic cancer tissue has the highest DCLK1 mRNA expression levels among the gastrointestinal cancer types (Figure 1(a)). In addition, we analyzed immunohistochemistry staining from the Human Protein Atlas generated using anti-DCLK1 antibody (Abcam 31704) that has been characterized by us and other groups extensively in the past [14, 23–25]. According to the Human Protein Atlas data, 100% of carcinoid, melanoma, colon, and breast and approximately 90% of glioma, pancreatic, and stomach cancer tissue expressed DCLK1. Notable expression (>50%) was also present in prostate, cervical, thyroid, endometrial, and lung cancer tissue (Figure 1(b)). DCLK1 expression in the normal pancreas is isolated to glandular exocrine cells, while it is overexpressed in both tumor epithelial and stromal cells in the cancer tissue (Figure 1(c)).

### 3.2. DCLK1 Expression in PDAC Normal Adjacent Tissue Predicts Poor Overall Survival

**Figure 2:**
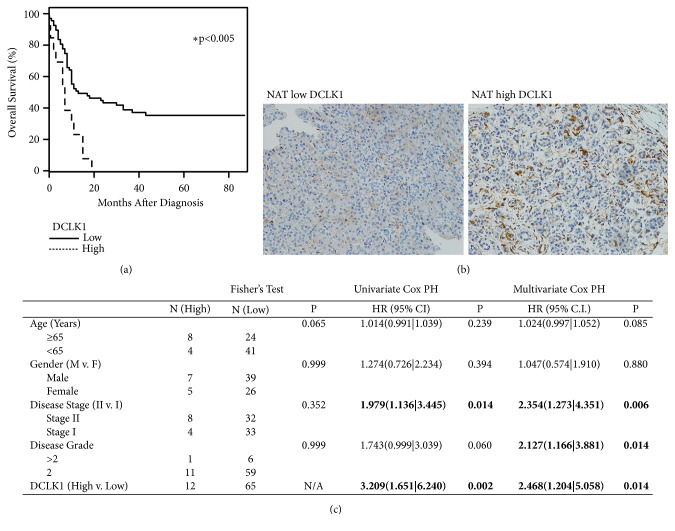
*High DCLK1 expression in normal adjacent tissue of PDAC correlates with poor survival*. (a) The intensity of DCLK1 expression in PDAC normal adjacent tissue (NAT) was scored based on staining using anti-DCLK1 antibody on a commercially available tissue microarray. Kaplan-Meier analysis of the DCLK1 staining scores demonstrated that patients with high levels of DCLK1 in the NAT had significantly shorter survival time compared to patients with low levels of DCLK1 in the NAT. (b) Representative images of low and high DCLK1 staining in NAT. (c) Multivariate Cox regression analysis of patients included in the TMA.

To further evaluate DCLK1 protein expression in PDAC tumors, we performed immunohistochemistry using anti-DCLK1 antibody on a commercially available tissue microarray with tumor and normal adjacent tissues (NAT) from stages I/II pancreatic cancer patients. We found higher expression of DCLK1 in most of the tumor samples and assessed the effect of DCLK1 expression on patient survival. The expression levels of DCLK1 in the tumor tissues did not predict survival (data not shown). However, patients with high levels of DCLK1 in the NAT had significantly reduced overall survival compared to patients with low levels (median 6-7 months and 12-13 months, resp.) (Figures 2(a) and 2(b)). Controlling for all other factors including age, gender, disease grade, and disease stage using multivariate Cox analysis confirmed this finding (Figure 2(c), p=0.014), suggesting that NAT DCLK1 may be an independent prognostic factor. These findings expand on previous findings demonstrating that tumor DCLK1 predicts survival in PDAC [26] and suggest a potential protumorigenic role for normal DCLK1+ cells adjacent to the tumor.

### 3.3. PDAC Patients Expressing DCLK1 Demonstrate PI3K/AKT/MTOR Pathway Activation

**Figure 3:**
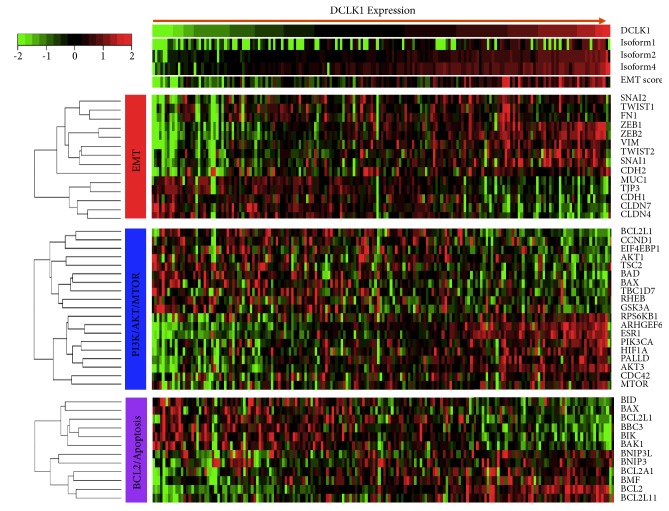
*PDAC patients expressing DCLK1 demonstrate PI3K/AKT/MTOR pathway activation*. RNA-Seq expression data from TCGA PAAD were analyzed. Patients were grouped based on DCLK1 expression and compared expression of genes downstream of RAS activation, grouped into EMT, PI3K/AKT/MTOR, and BCL2/Apoptosis.

In order to determine whether DCLK1 expression level correlates with KRAS related pathways in human PDAC patients, we analyzed RNA-Seq expression data from TCGA (PAAD). We grouped patients into DCLK1-low (bottom 25th percentile) and DCLK1-high (top 25th percentile) groups and compared expression of genes downstream of RAS activation. We found that DCLK1-AL and BL are associated with increased EMT based on genetic signature analysis. In addition, DCLK1-high patients have increased expression of PI3K/AKT/MTOR and downstream signaling pathways which support stemness, antiapoptosis, and tumorigenesis (Figure 3). Taken together, these findings support a role for DCLK1 in regulating KRAS-mediated pathway activation and confirm recent findings of DCLK1-associated PI3K/MTOR activity [26].

### 3.4. Overexpression of DCLK1-AL Increases PDAC Invasion, Drug Resistance, and KRAS Activation

**Figure 4:**
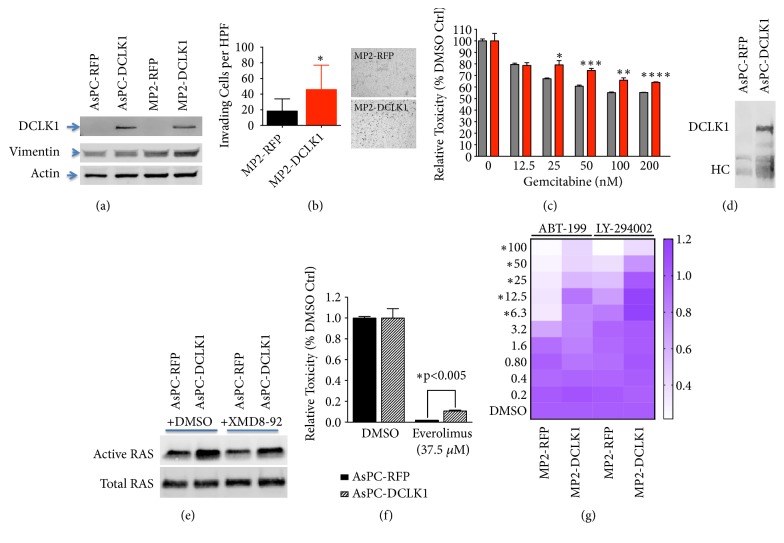
*Overexpression of DCLK1-AL in pancreatic cancer cells increases cell invasion, drug resistance, and KRAS activation*. (a) Both AsPC-1 and MP2 cells were infected with lentivirus containing either DCLK1-AL-RFP or RFP cDNA sequence to establish stable cell lines overexpressing DCLK1. (b) Matrigel coated transwell assays were used to study cell invasion activity. (c) Overexpression of DCLK1-AL increases pancreatic cancer cell resistance to Gemcitabine. (d) DCLK1-AL forms a complex with RAS. (e) Overexpression of DCLK1AL increases active RAS in pancreatic cancer cells. (f-g) Overexpression of DCLK1-AL increases pancreatic cancer cell resistance to Everolimus, ABT-199, and LY-294002.

To assess the effects of DCLK1-AL on PDAC, we established stable cell lines overexpressing DCLK1-AL-RFP fusion protein in AsPC-1 and MP2 cells using RFP as control (Figure 4(a)). The DCLK1-AL-RFP fusion protein was detected with anti-DCLK1 antibody, while endogenous DCLK1 protein level was barely detectable by western blot in these two lines (Figure 4(a)). To assess the effect of overexpressing DCLK1-AL on pancreatic cancer cell invasion, Matrigel coated invasion assays were performed. Overexpressing DCLK1-AL in MP2 cells increased cell invasion more than 2-fold (Figure 4(b), p<0.005) and increased Vimentin expression was also detected in MP2-DCLK1 cells (Figure 4(a)).

Drug resistance is a mechanism by which quiescent tumor stem cells maintain viability while the bulk of the tumor is destroyed by chemotherapies targeting rapidly dividing tumor cells. To assess whether overexpression of DCLK1-AL increases drug resistance, we treated MP2-RFP and MP2-DCLK1 cells with various concentrations of gemcitabine for 48 h and performed an MTT assay. MP2-DCLK1 cells significantly resisted gemcitabine treatment compared to MP2-RFP cells at most doses (p<0.05) (Figure 4(c)).

Using a coimmunoprecipitation assay, we found that DCLK1-AL forms a complex with RAS (Figure 4(d)) in DCLK1-AL overexpressing cells consistent with the findings reported by Westphalen et al. [9]. In order to assess whether DCLK1 regulates the activation of RAS, we performed an active RAS pull-down assay to detect the GTP-bound active form of RAS in AsPC-DCLK1 or AsPC-RFP cells following serum starvation and stimulation with FBS-containing media. DCLK1-AL overexpression resulted in an approximately 3-fold increase in active RAS (Figure 4(e)). In order to determine if this activation was regulated by DCLK1 kinase activity, we treated cells with DCLK1 kinase inhibitor XMD8-92 [20]. Treating AsPC-DCLK1 cells with XMD8-92 (15 *μ*M) for 15 min significantly inhibited the activation of RAS under these conditions. However, XMD8-92 treatment was unable to inhibit RAS-activation in AsPC-RFP cells expressing endogenous levels of DCLK1 (Figure 4(e)). These findings suggest that the use of DCLK1 kinase inhibitors may be beneficial in patients expressing high levels of DCLK1 by impairing RAS activation.

Since high DCLK1 expression in pancreatic cancer patients is correlated with activation of pathways downstream of RAS (PI3K/MTOR) (Figure 3), we also assessed the effect of overexpressing DCLK1-AL on Everolimus (MTOR inhibitor), LY-294002 (PI3K inhibitor), and ABT-199 (BCL-2 inhibitor) treated pancreatic cancer cells. AsPC-DCLK1 cells significantly resisted Everolimus (37.5 *μ*M) compared to control cells (p< 0.005) (Figure 4(f)), and MP2-DCLK1 cells significantly resisted both ABT-199 and LY-294002 compared to control cells at most doses (p<0.05) (Figure 4(g)). These findings suggest that DCLK1-AL overexpression is an important factor in PDAC drug resistance.

### 3.5. Anti-DCLK1 Monoclonal Antibodies Inhibit PDAC Tumorigenesis In Vivo

**Figure 5:**
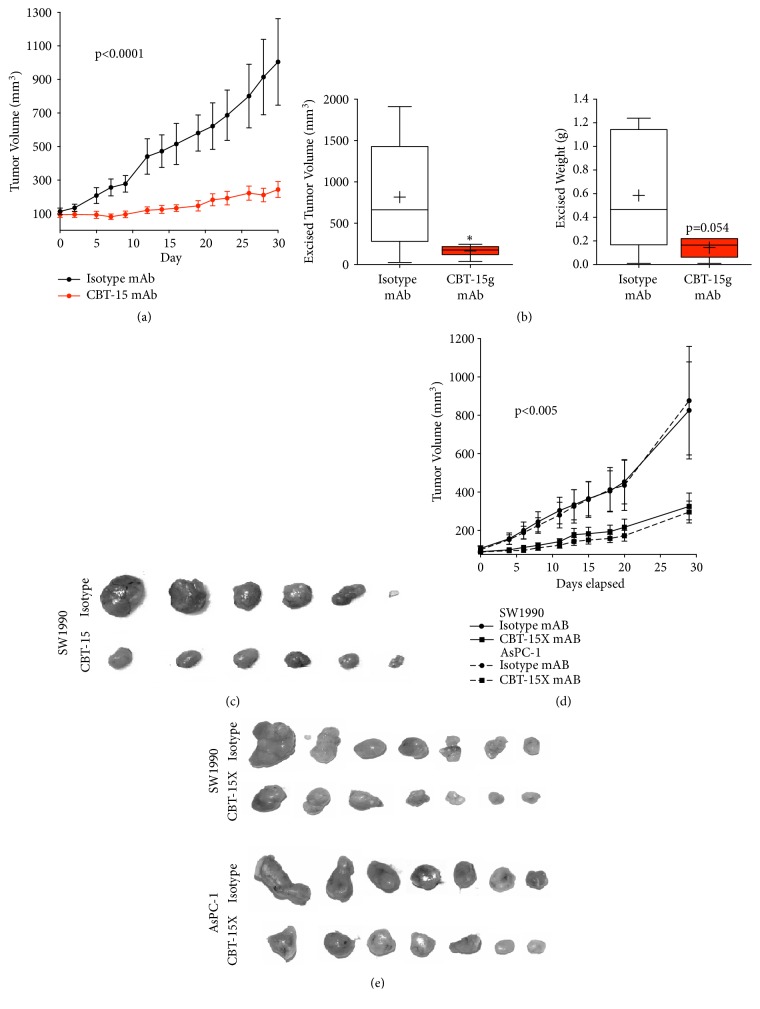
*Anti-DCLK1 mAbs inhibit pancreatic cancer xenograft tumor growth*. (a) Biweekly injection of CBT-15 mAb (*i.p.*) significantly impairs SW1990 pancreatic cells originated tumor xenograft growth (p<0.0001) as confirmed by (b) decreased excised tumor volume and (c) decreased excised tumor mass. (d) Biweekly injection of CBT-15X mAb (*i.p.*) significantly impairs SW1990 (solid line with solid squares) and AsPC-1 (dashed line with solid squares) pancreatic cells originated tumor xenograft growth (p<0.005) as confirmed by (e) decreased excised tumor mass.

We recently reported that monoclonal antibody CBT-15 targeting DCLK1-AL/BL inhibits renal cancer tumorigenesis* in vivo*. In order to evaluate the effect of targeting DCLK1 in pancreatic cancer tumorigenesis, we utilized a novel mAb (CBT-15G), which differs from CBT-15 which we recently reported in renal cell cancer [21]. To determine its effects on pancreatic cancer tumorigenesis, we established SW1990 pancreatic cancer cells xenografts in athymic nude mice. Upon reaching 100 mm^3^ average tumor volume, CBT-15G was delivered* i.p. *biweekly at 25 mg/Kg for 4 weeks and changes in tumor volume were assessed every other day. CBT-15G therapy dramatically reduced SW1990* in vivo *tumorigenesis over time, which was confirmed by assessing excised tumor volume and weight (Figures 5(a)–5(c)). Following confirmation of CBT-15G's* in vivo *efficacy, the variable region of the mAb was sequenced and a stable 293T cell line secreting the mouse-human chimera version of the mAb (CBT-15X) was generated. Following establishment of the line and purification of secreted CBT-15X, another set of xenografts were prepared as described for the mouse antibody for both SW1990 and AsPC-1 PDAC cell lines. Biweekly* i.p. *CBT-15X therapy also led to a marked, thorough decreased inhibition of* in vivo *tumorigenesis in these xenografts (Figures 5(d)-5(e) and Figure S1).

**Figure 6:**
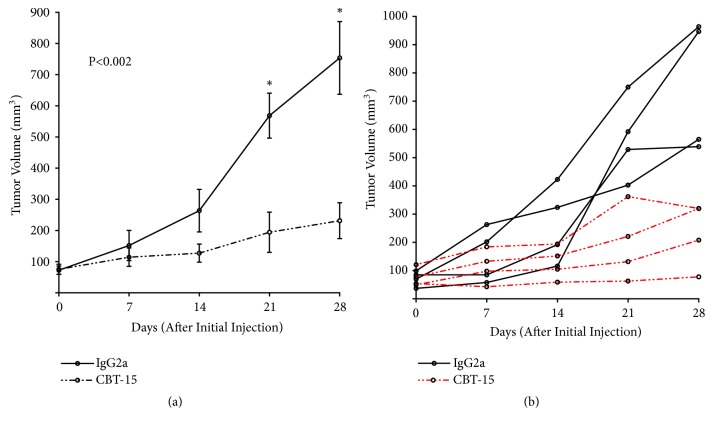
*Anti-DCLK1 mAb inhibits pancreatic tumor growth in KPC mice*. Biweekly injection of CBT-15 mAb (*i.p.*) significantly impairs pancreatic tumor growth (p<0.002) in KPC mice (n=4 in each group). (a) Average tumor volumes. (b) Individual tumor volumes.

Although athymic nude mice maintain a partially functional immune system, mAb therapies are best assessed in models with full immune function. Given our recent promising findings in renal cell cancer [21], we converted CBT-15 (an IgA) to both a mouse and fully humanized IgG. To test the activity of this antibody, we delivered it at 25 mg/Kg* i.p. *to KPC mice on a biweekly basis for 4 weeks (16-20 weeks of age). Tumor growth was tracked using ultrasonography. To assess tumor growth accurately, we selected mice with initial tumor sizes <100 mm^3^ and only assessed those (n=4 in each group) that survived the duration of the study. The antitumor activity of CBT-15 was clear based on both average and individual differences in tumor growth (Figure 6). In totality these findings provide the first proof of concept for DCLK1-targeted mAb therapy against pancreatic cancer.

## 4. Discussion

Despite advances in the understanding of pancreatic cancer biology and in surgical and medical therapy in recent years, little impact has been made on the mortality associated with this cancer. Therefore, there is an unmet need to find new therapeutic approaches against PDAC. Zhang et al. reported recently that DCLK1 levels in PDAC tumor tissues predict poor survival [26]; we also found that DCLK1 levels in PDAC NAT can predict poor survival; taken together, these studies suggest that DCLK1 levels could be used as a prognostic biomarker for PDAC.

There are two DCLK1 isoforms transcribed from the *α*-promoter, isoform 1 (*α*-short) and isoform 2 (*α*-long) [13]. It has been reported that overexpressing DCLK1 *α*-short in pancreatic cancer cells increased cell proliferation, migration, and invasion [27, 28]. In this study, we demonstrated that overexpressing DCLK1 *α*-long in pancreatic cancer cells also increases these functional properties and drug resistance. In our previous studies of DCLK1 *α*-long functionality in clear cell renal cancer, we found that its expression strongly supports stemness as determined by 3D spheroid assays, drug resistance assays, and expression of well-described stem cell markers [21]. Similar studies in pancreatic cancer demonstrate comparable results [9, 14, 28]. To assess the potential contribution of stemness to our results in this study, we performed a spheroid assay and found a three-fold increase in spheroid formation efficiency using MP2-DCLK1 cells compared to MP2-RFP cells, suggesting that overexpression of DCLK1-AL increases stemness (Figure S2).

KRAS activating mutations are present in 95% of PDAC tumors, but targeting KRAS directly has been unsuccessful so far and many inhibitors have failed in clinical trials [4]. Here we have confirmed previous studies demonstrating DCLK1 upregulation in PDAC. Importantly, we demonstrate for the first time that its upregulation directly increases the activation of KRAS, suggesting that it is a potential upstream activator. In addition, DCLK1 levels correlate with RAS downstream signaling effectors, PI3K and mTOR in RNA-Seq expression data. These findings offer a potential explanation for previous work showing DCLK1's ability to drive tumor proliferation, migration, and invasion. Functionally, the present study shows that cells overexpressing DCLK1 are resistant to standard doses of the FDA-approved inhibitors against PI3K and mTOR. In fact, approximately 50% more mTOR inhibitor Everolimus and 30% more PI3K inhibitor LY-294002 were required to inhibit cell proliferation. These findings suggest the potential benefits of targeting DCLK1 in these patients as a primary therapy or as a cotreatment with PI3K, MTOR, or EGFR-targeted drugs which have so far demonstrated insignificant efficacy in trials likely due to the high prevalence of KRAS mutations.

In order to evaluate the effect of targeting DCLK1* in vivo*, we utilized a novel mAb (CBT-15G) as well as a production-ready version of the mAb that we recently reported against DCLK1 in renal cell cancer [21]. Targeting DCLK1 with these mAbs in xenograft mouse models from KRAS^G12D^ mutant human cell lines AsPC-1 or SW1990 or in the KPC mouse model led to significant inhibition of the tumor growth (Figures 5 and 6). These data demonstrate that DCLK1-targeted mAbs or other targeted therapies may be effective against PDAC.

In summary, the studies reported here illustrate the role of DCLK1 in KRAS activation, PDAC tumor cell invasion, drug resistance, pancreatic tumor growth* in vivo*, and overall patient survival. Analysis of DCLK1 expression across tissue types demonstrates a favorable pattern for targeted cancer therapy. Moreover, it is notable that although DCLK1 is expressed in normal glandular/tuft cells, which play an important role in response to inflammatory injury [29–31], the available data demonstrates that knockdown or knockout of DCLK1 or deletion of DCLK1+ cells [9, 30, 32] does not result in undue toxicity or significantly impacts homeostatic conditions. In combination these findings suggest that targeting DCLK1 may have significant therapeutic potential and a low side-effect profile as a primary therapy or in conjunction with existing drugs.

## 5. Conclusions

DCLK1 promotes KRAS-driven PI3K/AKT/mTOR signaling in PDAC leading to increased invasive, antiapoptosis, stemness, and tumorigenic properties. DCLK1-targeted therapies may overcome this signaling and improve PDAC outcomes.

## Data Availability

The data used to support the findings of this study are included within the article.

## Supplementary Materials

Supplementary Materials Figure S1: CBT-15X chimeric mAb inhibits pancreatic cancer xenograft tumor growth. A. Excised tumor volume and tumor mass from SW1990 pancreatic cancer cells originated xenograft. B. Excised tumor volume and tumor mass from AsPC-1 pancreatic cancer cells originated xenograft. Figure S2: overexpression of DCLK1-AL in MP2 cells enhances tumor spheroid formation. A-B. Spheroids formation is significantly enhanced in MP2-DCLK1 cells (P<0.0001). C. Representative images display differences between MP2-RFP and MP2-DCLK1 spheroid formation. Table S1: Patient Characteristics. Publicly available, deidentified data were accessed from TCGA, and basic characteristics of the PDAC patients are presented. Table S2: Univariate and Multivariate Analyses. Publicly available, deidentified data were accessed from TCGA for the analysis reported here.
