# Supplementary material for: Overexpression of DCLK1-AL Increases Tumor Cell Invasion, Drug Resistance, and KRAS Activation and Can Be Targeted to Inhibit Tumorigenesis in Pancreatic Cancer
Source: J Oncol. 2019 Aug 5;2019:6402925. doi: 10.1155/2019/6402925 (PMC6699308; doi:10.1155/2019/6402925)
Supplement: Supplementary Materials — Figure S1: CBT-15X chimeric mAb inhibits pancreatic cancer xenograft tumor growth. A. Excised tumor volume and tumor mass from SW1990 pancreatic cancer cells originated xenograft. B. Excised tumor volume and tumor mass from AsPC-1 pancreatic cancer cells originated xenograft. Figure S2: overexpression of DCLK1-AL in MP2 cells enhances tumor spheroid formation. A-B. Spheroids formation is significantly enhanced in MP2-DCLK1 cells (P<0.0001). C. Representative images display differences between MP2-RFP and MP2-DCLK1 spheroid formation. Table S1: Patient Characteristics. Publicly available, deidentified data were accessed from TCGA, and basic characteristics of the PDAC patients are presented. Table S2: Univariate and Multivariate Analyses. Publicly available, deidentified data were accessed from TCGA for the analysis reported here. [file 6402925.f1.pdf]

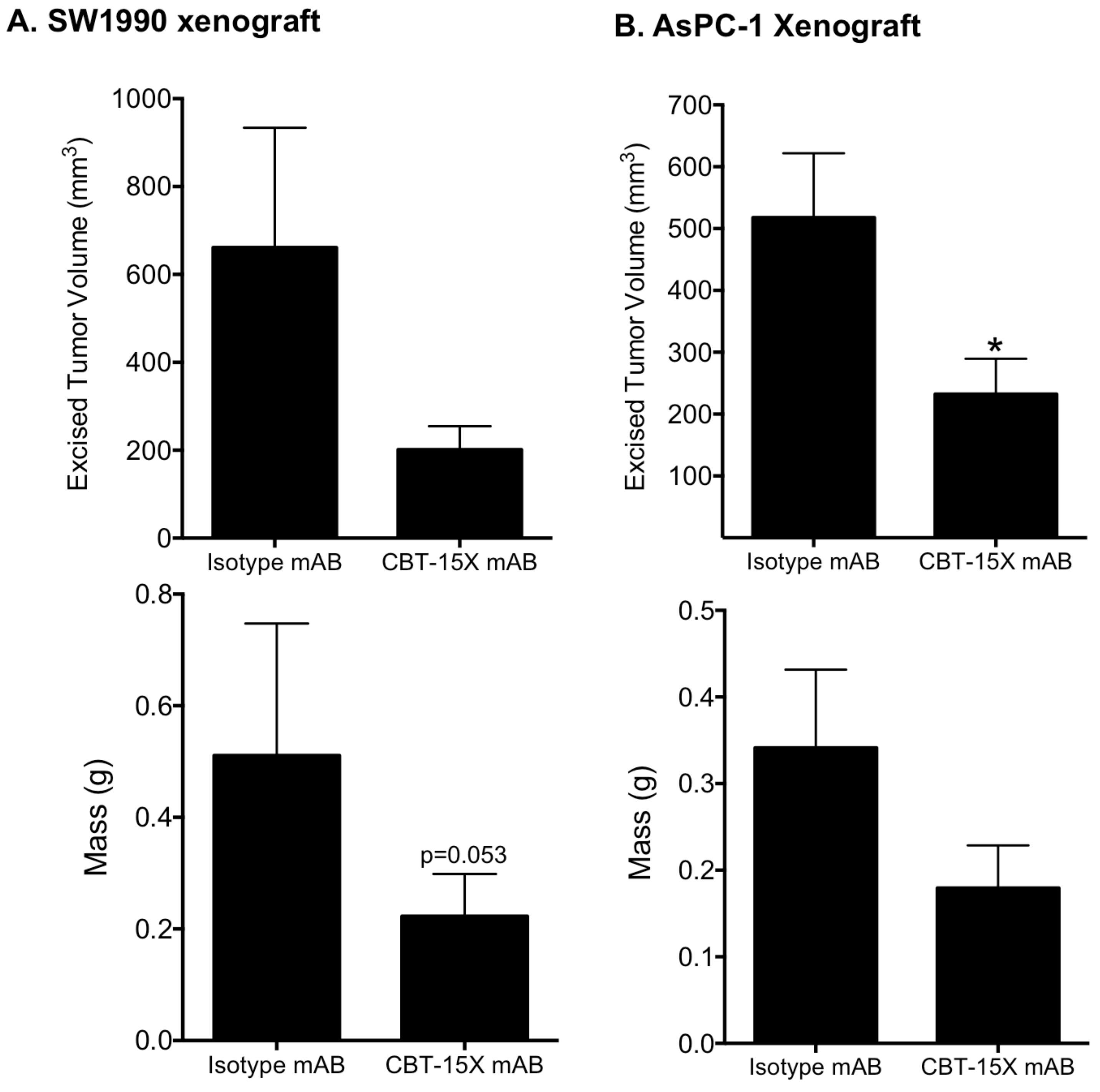

Supplementary Fig.1

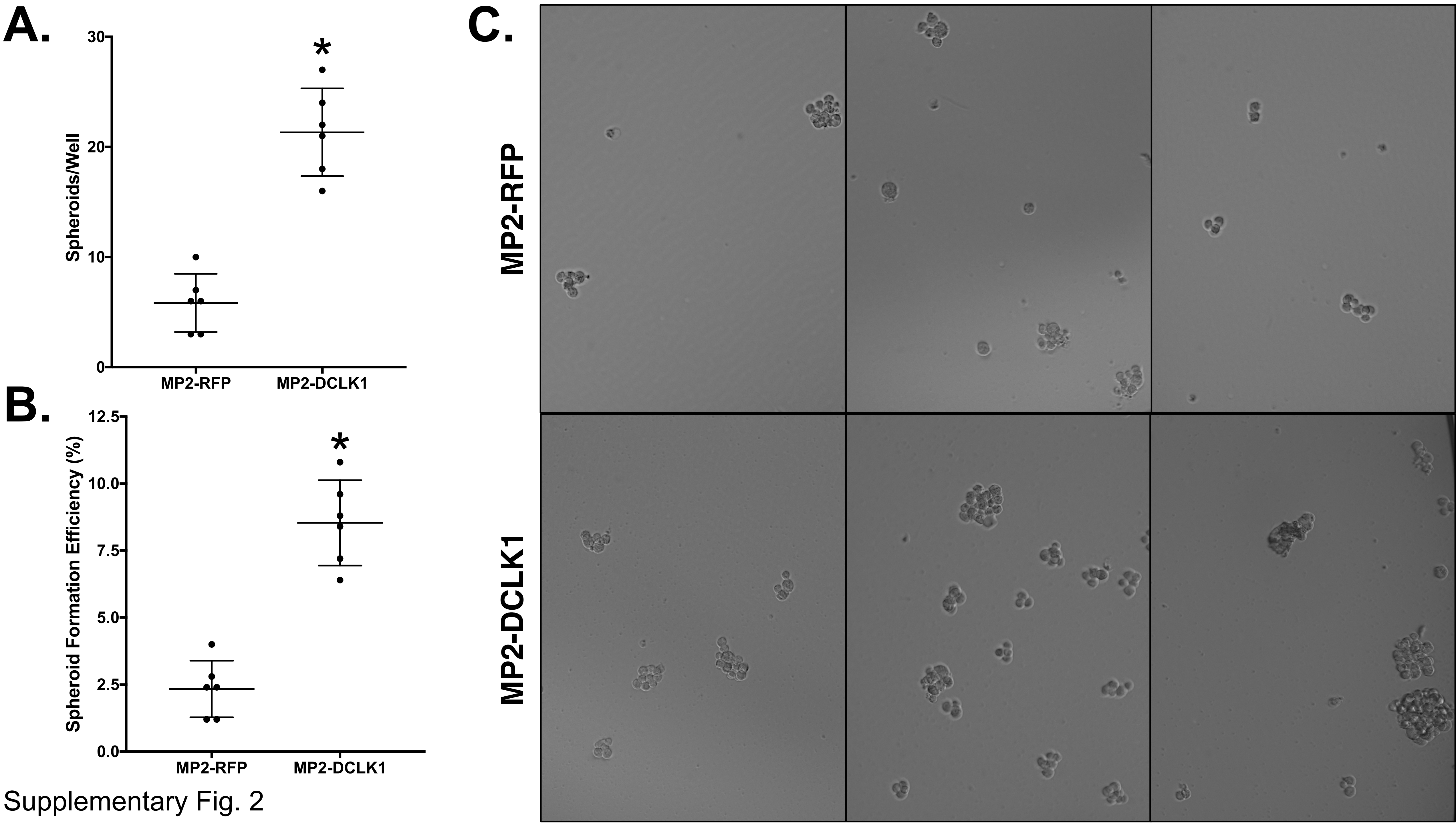

Supplementary Fig. 2

Supplementary Table S1. Patient characteristics.

Patient Characteristics

	N
All patients	185
Age	62
<65	91
>=65	94
Median(range)	65(35-88)
Gender	
Male	102
Female	83
Pathologic Stage	
Early(I+II)	173
Advanced(III+IV)	10
NA	2
Histologic Grade	
Low(G1+G2)	129
High(G3+G4)	53
NA	3
Metastasis	
Positive	5
Negative	85
NA	95
Node	
Positive	130
Negative	50
NA	5

Supplementary Table 2

	Univariate Analysis				Multivariate Analysis			
	HR(95% CI) OS	p	HR(95%CI)RFS	p	HR(95%CI)OS	p	HR(95%CI)RFS	p
Age(<65 vs >=65)	0.77(0.52|1.15)	0.203	1.09(0.64|1.83)	0.760				
Gender(Female vs Male)	1.20(0.81|1.78)	0.366	0.90(0.54|1.52)	0.704				
Tobacco History(Smoker vs Non-Smoker)	1.05(0.68|1.64)	0.815	1.35(0.76|2.39)	0.302				
Current Smoker vs Lifelong Non-Smoker	1.71(0.90|3.24)	0.098	0.96(0.36|2.57)	0.932				
Reformed Smoker vs Lifelong Non-Smoker	0.90(0.56|1.46)	0.682	1.46(0.81|2.65)	0.209				
Alcohol History(Drinker vs Non-Drinker)	1.12(0.73|1.73)	0.608	1.39(0.79|2.45)	0.257				
History of Diabetes(Present vs Absent)	0.93(0.54|1.62)	0.803	0.88(0.45|1.72)	0.706				
History of Chronic Pancreatitis(Present vs Absent)	1.19(0.57|2.48)	0.649	0.78(0.28|2.19)	0.637				
Family History of Cancer(Present vs Absent)	1.15(0.68|1.97)	0.598	0.87(0.45|1.69)	0.690				
Anatomic Subdivision(other parts vs Head of Pancreas)	0.55(0.30|1.00)	**0.048**	0.59(0.29|1.17)	0.127	0.61(0.28|1.30)	0.198		
Body of Pancreas vs Head of Pancreas	0.39(0.15|0.95)	**0.031**	0.40(0.14|1.11)	0.069				
Tail of Pancreas vs Head of Pancreas	0.76(0.35|1.65)	0.489	0.82(0.35|1.93)	0.649				
Histologic Grade(High vs Low)	1.56(1.03|2.36)	**0.036**	1.83(1.06|3.16)	**0.028**	1.49(0.92|0.51)	0.104	2.17(1.21|3.90)	**0.009**
Residual Tumor(With Tumor vs Tumor Free)	1.56(1.02|2.40)	**0.040**	1.85(1.05|3.26)	**0.031**	1.56(0.97|2.50)	0.065	2.04(1.15|3.62)	**0.015**
Node(Positive vs Negative)	2.17(1.31|3.59)	**0.002**	1.72(0.96|3.11)	0.069	2.51(1.38|4.54)	**0.002**		
Additional Pharmaceutical Therapy(Treated vs Untreated)	0.66(0.32|1.34)	0.242	1.22(0.69|2.17)	0.491				
Targeted Molecular Therapy(Treated vs Untreated)	0.47(0.31|0.72)	<**0.001**	1.11(0.59|2.07)	0.696	0.31(0.19|0.51)	<**0.001**		

Figure S1. CBT-15X chimeric mAb inhibits pancreatic cancer xenograft tumor growth. A. excised tumor volume and tumor mass from SW1990 pancreatic cancer cells originated xenograft. **B.** excised tumor volume and tumor mass from AsPC-1 pancreatic cancer cells originated xenograft.

Figure S2. Overexpression of DCLK1-AL in MP2 cells enhances tumor spheroid formation. A-B. Spheroids formation is significantly enhanced in MP2-DCLK1 cells ($P<0.0001$). C. Representative images display differences between MP2-RFP and MP2-DCLK1 spheroid formation.
